# Bottleneck Detection in Modular Construction Factories Using Computer Vision

**DOI:** 10.3390/s23083982

**Published:** 2023-04-14

**Authors:** Roshan Panahi, Joseph Louis, Ankur Podder, Colby Swanson, Shanti Pless

**Affiliations:** 1 School of Civil and Construction Engineering, Oregon State University, Corvallis, OR 97331, USA; 2 Research Engineer, National Renewable Energy Laboratory (NREL), Golden, CO 80401, USA; 3 Momentum Innovation Group, Jersey City, NJ 07302, USA

**Keywords:** modular construction, computer vision, bottleneck detection, deep learning, sensors

## Abstract

The construction industry is increasingly adopting off-site and modular construction methods due to the advantages offered in terms of safety, quality, and productivity for construction projects. Despite the advantages promised by this method of construction, modular construction factories still rely on manually-intensive work, which can lead to highly variable cycle times. As a result, these factories experience bottlenecks in production that can reduce productivity and cause delays to modular integrated construction projects. To remedy this effect, computer vision-based methods have been proposed to monitor the progress of work in modular construction factories. However, these methods fail to account for changes in the appearance of the modular units during production, they are difficult to adapt to other stations and factories, and they require a significant amount of annotation effort. Due to these drawbacks, this paper proposes a computer vision-based progress monitoring method that is easy to adapt to different stations and factories and relies only on two image annotations per station. In doing so, the Scale-invariant feature transform (SIFT) method is used to identify the presence of modular units at workstations, and the Mask R-CNN deep learning-based method is used to identify active workstations. This information was synthesized using a near real-time data-driven bottleneck identification method suited for assembly lines in modular construction factories. This framework was successfully validated using 420 h of surveillance videos of a production line in a modular construction factory in the U.S., providing 96% accuracy in identifying the occupancy of the workstations and an F-1 Score of 89% in identifying the state of each station on the production line. The extracted active and inactive durations were successfully used via a data-driven bottleneck detection method to detect bottleneck stations inside a modular construction factory. The implementation of this method in factories can lead to continuous and comprehensive monitoring of the production line and prevent delays by timely identification of bottlenecks.

## 1. Introduction

Off-site and modular construction is increasingly being seen as a promising method of project delivery due to the advantages offered in terms of productivity and cost [1]. It also enables the incorporation of energy-efficient building strategies at scale to reduce the initial cost of installation for affordable housing. Building components are produced in factory settings and shipped for site installation [2]. The highest degree of modularization is obtained in volumetric construction, where three-dimensional modular units are built off-site [3]. Despite these advantages, modular factories are heavily reliant on manual labor, which leads to bottlenecks on the factory floor caused by cycle time variability. These bottlenecks have been estimated to account for up to 15% of work time [4]. In effect, these bottlenecks significantly reduce the productivity and throughput of the factory and cause delays in modular integrated construction projects [5].

With the recent advancements in computer vision and the widespread use of cameras in construction projects, computer vision-based monitoring has attracted interest from the construction community at large [6]. However, research on the application of computer vision for progress monitoring inside modular construction factories is less mature and limited to applications such as identifying workers’ activities [7] and monitoring single workstations [8]. Specifically, previous progress monitoring methods were limited to a single station; they assumed the stations were already occupied, which is not true on the assembly lines. Previous methods relied on computationally expensive deep learning detection methods for all tasks that require a large number of annotations as the appearance of the modules changes during production. This paper addresses these drawbacks by proposing a vision-based method to identify the occupancy of the stations. Furthermore, this paper uses a template-based-matching approach which is more suitable for such a dynamically changing assembly line, and uses SIFT with less computational complexity and more data efficiency as it only relies on two annotations per station. 

Overall, this study proposes a practical computer vision-based method to identify the bottleneck station on the production line of modular construction factories. In doing so, a novel hand-crafted-based computer vision-based method was developed to identify the occupancy stations in modular construction factories, and a deep learning-based computer vision method was used to innovatively identify the state of each workstation. This information was synthesized using a novel and practical bottleneck identification formulism suited for the assembly line of modular construction factories to identify the bottlenecks in near real-time on the factory floor. 

The remainder of this manuscript is organized as follows: first, the state-of-the-art research in automated progress monitoring methods in modular construction, along with commonly-used bottleneck detection methods in the modular construction and manufacturing industry, is provided to the stage for the methodology developed in this paper. This is followed by a demonstration of implementing the proposed method in a volumetric modular construction factory in the U.S. Finally, the results and the conclusions of the research are presented and discussed.

## 2. Literature Review

This section presents an overview of automated monitoring research in off-site and modular construction factories and different bottleneck detection methods. Subsequently, the existing gaps in knowledge are delineated to set the context for the presented research.

### 2.1. Automated Work Progress Monitoring in Off-Site and Modular Construction

Monitoring the progress of work is an important aspect of effective project management [9]. The current state of practice for progress monitoring inside modular construction factories is highly reliant on manual progress monitoring procedures, which are time-consuming, laborious, and error-prone [10,11,12]. Therefore, researchers have sought different approaches to automate this process using various sensors to collect the work progress data. 

Previous research in automated progress monitoring for off-site and modular construction can be categorized into two groups based on the proximity of the sensor to the object of interest: (1) Contact sensor-based methods and (2) Remote sensor-based methods. These methods are described in more detail in the following subsections.

#### 2.1.1. Contact Sensor-Based Methods

In this category, the progress of work is monitored by manually attaching the sensors such as Global Positioning System (GPS), inertial measurement units (IMU), radio-frequency identification (RFID), and quick response (QR) tags to material, labor, and equipment for purposes of tracking their location and state. Zhong et al. [13] used RFID sensors to track the material inside the modular factory and GPS to track the arrival and departure of trucks outside. Khandakar et al. [14,15] used RFID and IMU to track the movement of modular units across the production line inside the factory. Altaf et al. [16] used RFID technology to monitor the productivity of the assembly station by tracking the walls in a prefabrication factory. 

Despite the accuracy obtainable by these methods, the following drawbacks hinder their practical application: they require the sensor to be installed on the resource, which can interrupt work processes [17]; they are highly susceptible to different sources of noise and the quality of the data is dependent on the post-processing methods [17]; a secondary source of data, such as a camera, is required to validate the data; and finally, sensors such as GPS do not perform well for indoor applications. Remote-sensor methods overcome these drawbacks, as discussed in the next section. 

#### 2.1.2. Remote Sensor-Based Methods

Remote sensor-based methods refer to methods that do not require the sensor to be in contact with the resource being tracked and include methods such as light detection and ranging (LiDAR), audio, and computer vision. Laser scanning technologies such as LiDAR are widely discussed in construction progress monitoring research [18,19], while their application in modular construction has been mostly limited to quality control [20,21]. LiDAR-based methods are expensive to deploy and require a significant amount of post-processing. Audio sensors have also been used to identify worker activities in modular factories [22]. However, this method is not sufficiently developed to handle multiple sources of sound simultaneously and can only be applied to a limited subset of worker activities. These limitations can be overcome using computer vision-based progress monitoring, which is the focus of this paper and will be described in the next section. 

##### Computer Vision-Based Methods

Computer vision means and methods refer to sensors and a combination of theoretical principles and algorithms which aim to autonomously replicate some of the tasks the human visual system performs [23]. The availability, low cost, and ease of use without intruding on work progress make these methods ideal for the continuous monitoring of construction projects [24]. 

The application of computer vision in modular construction factories has only recently gained significant interest and coincides with both the advancements achieved in computer vision and the growing appeal of modular construction. In this area, Chu et al. [25] used posture recognition to replicate a worker’s posture inside a virtual space and assess the ergonomic effect of the common tasks performed by workers inside the factory. Panahi et al. [7] used computer vision methods to identify the activities of the workers from the videos. Bao et al. [26] used instance segmentation to detect and track workers inside the factory and overcome the problem of occlusions. However, these methods mostly focus on stand-alone tasks such as detection and tracking instead of progress monitoring. 

To monitor the on-site installation progress of modular units, Zheng et al. [27] proposed a framework that extracts the cycle time for the installation of modules after being delivered to the construction site. They fine-tuned a Mask R-CNN object segmentation algorithm on a total of 1100 synthetic and real images of finished prefabricated modules. Although their research is conducted to monitor the on-site installation, the generated datasets can be used to monitor the work inside modular factories as well. In order to monitor the progress of work in panelized modular construction factory workstations, Martinez et al. [8] proposed a vision-based monitoring method that detects the crane and the workers in a single station and updates the parameters of a finite state machine to track the progress of work. However, their scope of work is limited to a single station, does not provide the information needed to identify the bottlenecks on the production line, assumes the station is already occupied and work has started, and is not adaptable to changes in the layout of the factory. More recently, Park et al. [28,29] created a synthetic image dataset of modular units inside the factory and evaluated a CNN-based 3D reconstruction network from the collected 2D synthetic images. Although precisely recreating the digital twin of the factory can be beneficial in applications such as quality control, this is an expensive option in practice in cases where single-view methods can be applied, such as using surveillance cameras to monitor the installation progress and updating the digital twin.

The synthesis of the conducted review of the literature shows that previously proposed applications of computer vision inside modular construction factories are limited in breadth and depth. Specifically, the scope of the previously proposed vision-based progress monitoring methods is limited to a single station and stand-alone computer vision tasks such as detection and tracking; previously proposed methods are not adaptable to changes in the factory layout, and they are not robust to significant changes in the visual appearance of the modular units; finally, they require a large number of annotations, and they overlook the bottleneck detection problem as a major application for progress monitoring methods. These drawbacks create a significant gap in the current body of knowledge related to automated progress monitoring inside modular construction factories and impede the application of such methods in practice. 

This study attempts to fill these gaps by widening the scope of monitoring to multiple stations; using a combination of traditional and deep learning-based methods; incorporating expert knowledge at the operational level to improve the adaptability and robustness of the method, relying on a minimum number of annotations; and integrating with a popular and commonly used bottleneck detection method in manufacturing. 

### 2.2. Bottleneck Detection Methods

Bottlenecks are congestion points in a production system [30], and they have the largest effect on slowing down or stopping the entire system [31]. Theory of Constraints [32] recommends that bottlenecks have to be detected and eliminated in order to improve the throughput. Bottleneck detection methods can be categorized into simulation and data-driven methods.

Simulation-based methods in modular factories have been previously used to model the entire or a portion of the production line and to forecast the bottleneck station using the wait duration [33]. However, such static models can become obsolete if the input parameters deviate from the initial distribution [34]. Recently, dynamic data-driven simulation models have been proposed to overcome this drawback by updating their parameters based on the progress of work [16,35]. However, these models are mostly used for forecasting and strategic planning rather than real-time monitoring and control. 

Data-driven methods can detect the bottleneck without building an analytical or a simulation model, using only the real-time data captured from the manufacturing systems [36]. Roser et al. [37] proposed a method to identify the bottleneck station by averaging the active time of each workstation during the observed period. Li et al. [38] proposed the Turning Point method, which counts the number of starved and blocked stations before and after each workstation and detects the bottleneck when this trend changes. More recent attempts in bottleneck detection research expand the application of these simple, practical, and well-recognized methods to real-time bottleneck detection [39] and applications in more complex production systems [40], some of which can be applied to modular construction factories as well. 

Due to the advantages offered by data-driven methods, this study attempts to extend the application of data-driven methods to modular construction factories in order to identify the bottlenecks in real time. Therefore, in this study, the method proposed by Roser et al. [37] is used to detect the bottleneck in modular construction factories using the extracted data from the proposed progress monitoring method, to better bridge the gap identified and discussed in the next section. 

### 2.3. Gaps in Knowledge and Point of Departure of the Research 

Based on the literature review conducted, the following specific research gaps are identified and addressed in this paper:

(a) Unfeasibility of contact-based monitoring systems in modular construction factories: Majority of the previously proposed monitoring methods inside modular construction factories rely on contact-based sensors. Despite the accuracy these methods provide, they are expensive to implement at large scale, susceptible to noise, and intrusive to the progress of work. They also require validation using a secondary video data source during the data training phase.

(b) Assumption of workstation occupancy: Previously proposed vision-based monitoring methods assume the workstations are already occupied. However, identifying the occupancy of the stations is a major step in monitoring the progress of work, especially for the stations located on the assembly line where modular units move from one station to the next during the production process. 

(c) Limited application of vision-based progress monitoring: The scope of the computer vision-based progress monitoring methods in previous research is limited to a single workstation which introduces two main gaps: (1) In order to identify the bottlenecks, vision-based methods have to be proposed to simultaneously monitor multiple workstations at a larger scale inside the factory, and (2) Monitoring the progress of work at a larger scale, exposes the computer vision algorithms to additional challenges such as significant changes in the appearance of the modules, from a flat floor to a full volumetric unit, especially in the case of volumetric modular construction, which has not been addressed before. 

(d) Lack of a vision-based bottleneck detection framework for modular construction factories: Previous research has proposed stand-alone methods to extract progress data from the modular construction factory floor; however, they fall short in synthesizing this information towards a suitable bottleneck detection framework for modular construction factories. In addition, stations in these factories become inactive multiple times during a single cycle which can affect the bottleneck stations. However, this effect has not been considered in identifying the bottlenecks in previous vision-based monitoring methods in modular construction factories. 

(e) Inability of existing methods to adapt to changes in the factory floor: Previously proposed progress monitoring methods fall short in adapting to the changes in the factory layout as they attempt to propose fully automated solutions for monitoring the progress of work. However, it is extremely difficult for automated systems to identify changes in the layout and logic of production. Contrarily, incorporating the knowledge of experts in the monitoring system at the operation stage can reduce the cost of application and make the system robust to such changes. 

Considering the above-mentioned gaps in knowledge, the goal of this research is to develop a practical computer vision-based progress monitoring method and identify the bottleneck workstation in modular construction factories. In doing so, this paper relies on fundamental bottleneck detection theories and attempts to propose a new computer vision-based method to extract their required parameters from CCTV video footage of the production line, including multiple workstations. 

The next section is dedicated to the developed methodology to address the mentioned gaps and identify the bottleneck workstation in modular construction factories using the proposed automated vision-based progress monitoring method.

## 3. Methodology

In this section, the scope of application of the developed methodology is first described to delineate the type of factories and assembly lines to which the developed methodology can be applied. This is followed by a description of the proposed framework. 

### 3.1. Scope of the Methodology

This study is focused on the assembly line of volumetric construction factories. The typical layout of a modular construction factory consists of subassemblies such as walls and roofs, built in different workstations and transferred to the assembly line, where they are installed on the modular units as they move from one station to the next. The time spent by the module at each workstation on the assembly line is defined as ‘cycle-time’. It is assumed that this assembly line is buffer-less; therefore, delays at any of the workstations in this region can cause bottlenecks and impact the entire production. This makes this portion of the factory a critical section for the entire production line and the proposed method empirical to modular construction factories. 

### 3.2. Proposed Method 

**Figure 1:**
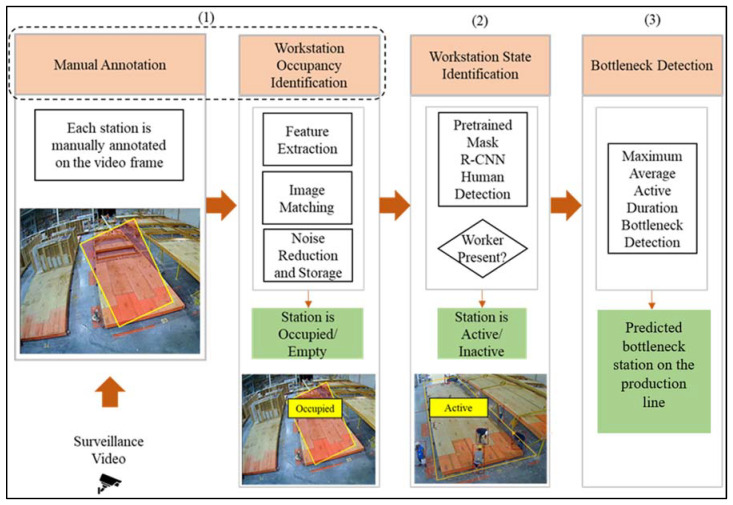
Proposed progress monitoring and bottleneck detection method.

The goal of this research is to automatically identify the bottleneck station on the assembly line of modular construction factories using computer vision. Figure 1 shows the overview of the objectives, methods used, and expected outcomes to achieve this goal. In this figure, the color orange denotes the objectives of this research, and the color green denotes the expected outcome, followed by a depiction of expected results for each objective. 

As shown in Figure 1, the objectives of this study are: (1) workstation occupancy identification: to identify if the workstation is occupied by a modular unit and classify the station as occupied or empty; (2) workstation state identification: to identify if workers are present at the workstation and classify the station as active or inactive, and (3) bottleneck detection: to identify the bottleneck workstation.

#### 3.2.1. Workstation Occupancy Identification 

This objective aims to monitor the progress of work on the assembly line by timestamping when the modular units enter and leave the workstation. This information can later be used to estimate the cycle time for each station. After the regions of interest are manually annotated on the video frame, keypoints of the annotated regions are extracted; image matching is used to classify the station, and noise is reduced in favor of the majority classes. These steps are described in more detail in the following section. 

##### Manual Annotation

**Figure 2:**
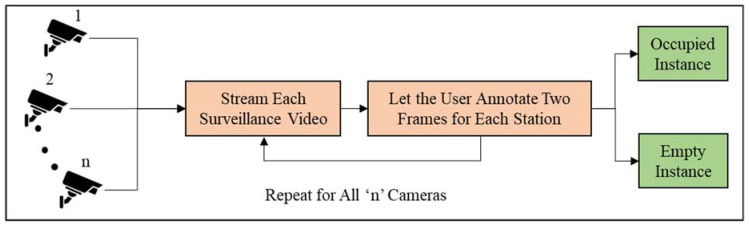
Manual annotation pipeline.

The location of each station is manually annotated on the video frame using Tkinter graphical user interface package in Python. Figure 2 shows the manual annotation pipeline. 

As shown in Figure 2, the user is required to annotate two instances for each station based on whether there is a modular unit present in the Region of Interest (RoI) or not. Subsequently, two ground-truth RoIs are created for each station and will be used to classify the station in the rest of the video as occupied or empty. Thus, n stations require 2×n ground truth RoIs. This pipeline is shown in Figure 2. 

This approach enhances the contribution of this study to the body of practice by: (1) reducing the cost of annotation: this method only requires 2 × n ground truth images, which is significantly less than the size of datasets required for deep learning-based detection methods, and (2) reducing the number of miss-classifications: the appearance of the modular units significantly changes during the production, which can pose challenges to computer vision-base methods. This method uses different ground-truth images per station, which limits the variability of images that occur during the progress of work, therefore, reduces the number of miss-classifications. 

##### Feature Extraction

**Figure 3:**
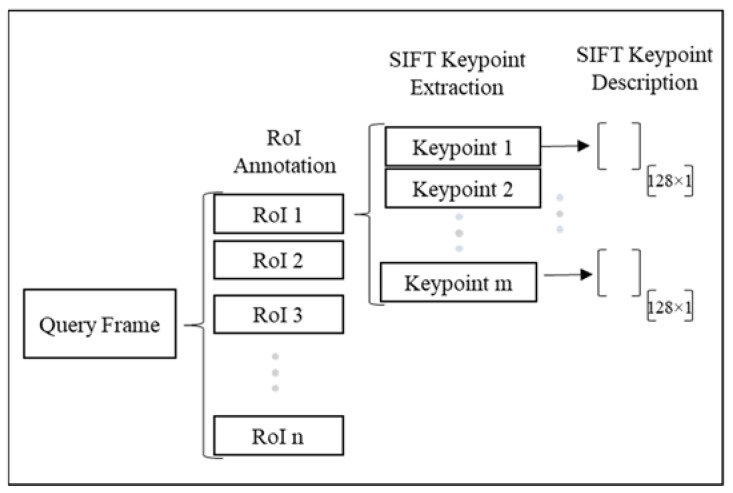
Feature extraction for ground truth and the rest of the video.

Scale Invariant Feature Transform (SIFT) [41] is used to detect and describe the keypoints in each image. In this case, since the modular units move along the production line and with respect to the camera, SIFT is the preferred choice for feature extraction since it is invariant to scale changes. Figure 3 shows the feature extraction pipeline for a sample query image. 

As shown in Figure 3, m keypoints are detected in each region of interest and described by computing a vector of size 128 for each keypoint. 

##### Image Matching 

**Figure 4:**
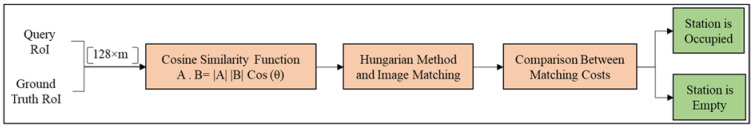
Image matching and occupancy identification pipeline.

Using features extracted from the previous step, the regions of interest are compared to the ground truth and assigned an empty or occupied state using image matching. Figure 4 shows how the query regions of interest are compared with the ground truth in three steps. Here, the letter ‘m’ indicates the number of keypoints detected in each RoI or ground truth image.

As shown in Figure 4, Cosine Similarity Function is used to estimate the similarity between the images, and the Hungarian method [42] is used to assign the best matches for each keypoint. 

##### Noise Reduction and Storage

**Figure 5:**
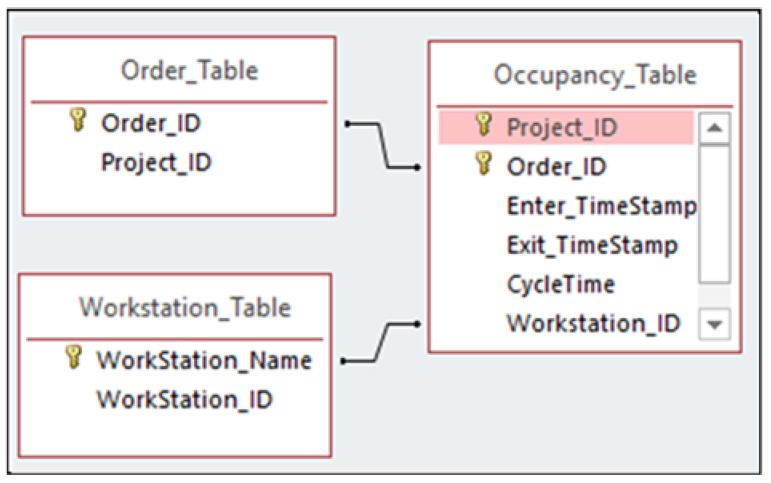
Relational database storing the state of workstations.

Applying the proposed method to the entire video may result in a few misclassifications. To remedy this effect, after the method has been evaluated, noise is reduced using the Median Filter method. Median Filter is a nonlinear smoothing method where a window is slid over the input replacing each input with the median of the inputs in the filter. The result is stored in a relational database shown in Figure 5. 

As shown in Figure 5, the raw data are parsed into structured data and stored in Boyce–Codd third normal form in Microsoft Access. In this figure, each modular unit can be identified using the project ID, where the information regarding the cycle time data can be accessed for each station.

#### 3.2.2. Workstation State Identification

**Figure 6:**
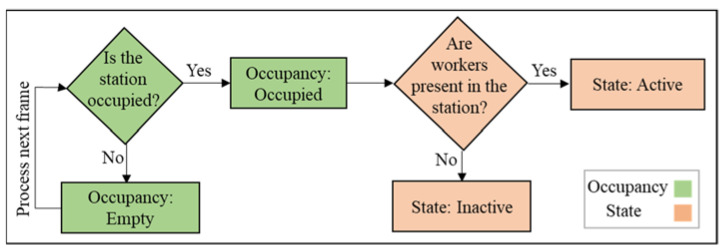
Workstation identification pipeline for occupied workstations.

Occupied workstations can be in active or inactive states during the cycle time. Due to the labor-intensive nature of the tasks, it is assumed that the workstation is active when it is occupied by a modular unit and workers are present, and it is inactive when it is occupied and workers are not present. Figure 6 shows how the state of each workstation is identified after the occupied station is detected. 

**Figure 7:**
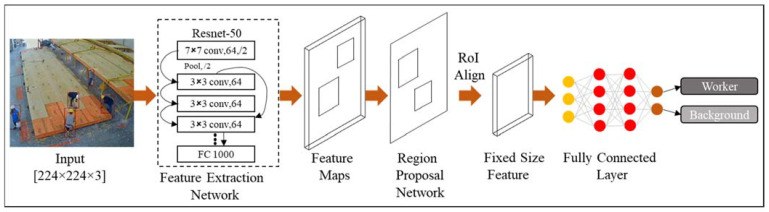
The architecture of the Mask R-CNN detection algorithm used in this study.

To automatically identify the state of workstations, the Mask Region-based Convolutional Neural Network (Mask R-CNN) [43] object detector with Resnet-50 backbone is used to detect the workers inside the workstation. Transfer learning is used by pretraining the model on the MS-COCO publicly available dataset [44]. Figure 7 shows the architecture of the employed deep learning-based detection algorithm.

As shown in Figure 7, the resized RGB video frame is input to the Mask R-CNN model. The resent-50 network with the ReLU activation function extracts the features of the input image using fifty convolutional neural network layers. These features are stacked into a feature map and used to generate object proposals using the Region Proposal Network module. These object proposals are then passed through a series of RoI (region of interest) pooling layers, which are designed to extract fixed-sized feature maps from each proposal. Finally, the fully connected layer is used to classify the regions to workers or backgrounds. 

The result from the implementation of this model is a bounding box drawn around the workers detected in the image. The instances where the center of the bounding box falls out of the annotated stations are discarded in order to identify the occupancy of the stations. In order to discard the random walks which occasionally take place on the shop floor, the station is considered occupied only if at least one worker exists inside the annotated bounding box of the station for the majority of the one-minute duration. With the state and occupancy of the workstation identified, the next objective is to identify the bottleneck station, as explained in the next section. 

#### 3.2.3. Bottleneck Detection 

**Figure 8:**
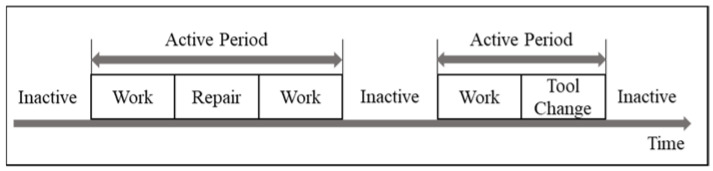
The active period of a machine during production.

This study uses the average active time method [37] to detect the bottleneck stations. This method is independent of the manufacturing system structure [45], can be easily automated [37], and applies to discrete event systems consisting of one or more workstations. According to this method, the bottleneck in a buffer-less production system similar to the one in this study is the workstation with the longest average active period. Figure 8 shows the active and inactive periods as defined in a typical factory.

**Figure 9:**
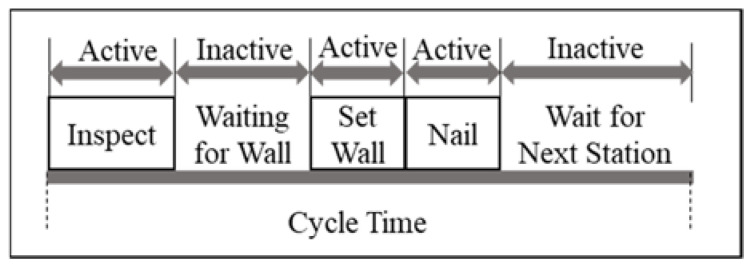
Active periods in a wall-set workstation during a single cycle time.

As shown in Figure 8, an active state in the manufacturing literature is the state of the machine when the machine produces a part or is being serviced or setup, whereas the inactive state of the machine is when the machine is waiting for the part or waiting to be serviced or waiting for the removal of the parts from the machine [33]. Figure 9 shows instances of active and inactive periods in the context of modular construction factories during the cycle time of each workstation. 

In Figure 9, performing inspection, wall-set, and nailing are considered active periods. and waiting for wall components and waiting for the next station are considered inactive periods. Due to the labor-intensive nature of tasks in modular construction factories, this paper assumes all active periods are associated with the presence of the workers. Table 1 shows more instances of the active and inactive periods that take place in modular construction factories.

As shown in Figure 9, the workstations can become inactive multiple times during each cycle time. Such interruptions can be prevalent in modular construction factories due to relatively long cycle times in workstations, which can be more than three hours [46]. This results in a set of durations A_i_ for each workstation i, as shown in Equation (1).

(1) $$A_{i}=\left\{a_{i,1},\;a_{i,2},\;\ldots a_{i,j},\ldots a_{i,n}\right\}$$

(2) $${\bar{a}}_{i}=\frac{\sum_{j=1}^{n}a_{i,j}}{n}$$

As shown in Equation (2), the average active duration ${\bar{a}}_{i}$ is calculated by taking an average of all active durations within each cycle time. Finally, the workstation with the longest average active period is considered to be the bottleneck since this workstation is least likely to be interrupted by other workstations, and in turn, it is the most likely to dictate the overall system throughput.

## 4. Case Study

**Figure 10:**
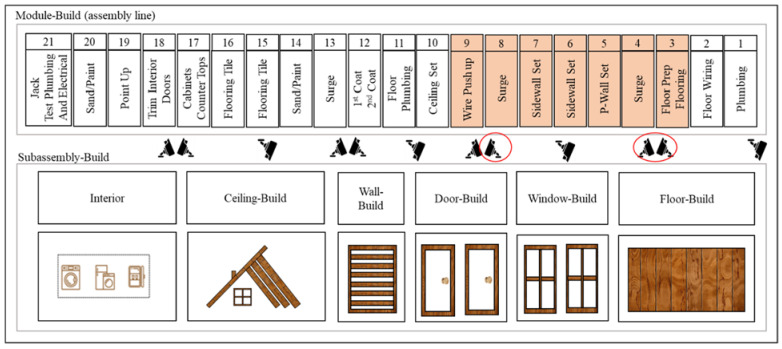
The layout of the surveillance cameras inside the factory.

The developed method was validated on the surveillance videos recorded from a wood frame-based volumetric modular construction factory in the US. Figure 10 shows the layout of this factory.

The goal of this section is to validate the developed method by monitoring the progress of work and identifying the occupancy and state of each workstation, and detecting the bottleneck station among the highlighted stations in Figure 10. As shown in this figure, the factory comprises 21 workstations on the assembly line and multiple workstations allocated to building floors, walls, ceilings, etc., in the subassembly-build section. The assembly line is monitored with surveillance videos where three camera views covering stations three to nine were selected for the purpose of this study. In the next section, the implementation of the developed method on the videos captured from these stations is explained in further detail.

### 4.1. Workstation Occupancy Identification 

#### 4.1.1. Data Collection and Manual Annotation

**Figure 11:**
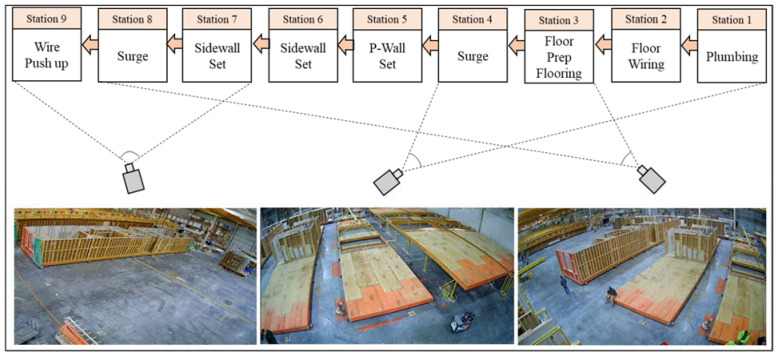
Surveillance camera view.

Video footage from three surveillance cameras covering the production line was collected. Figure 11 shows the camera installation layout with respect to the assembly line. 

**Figure 12:**
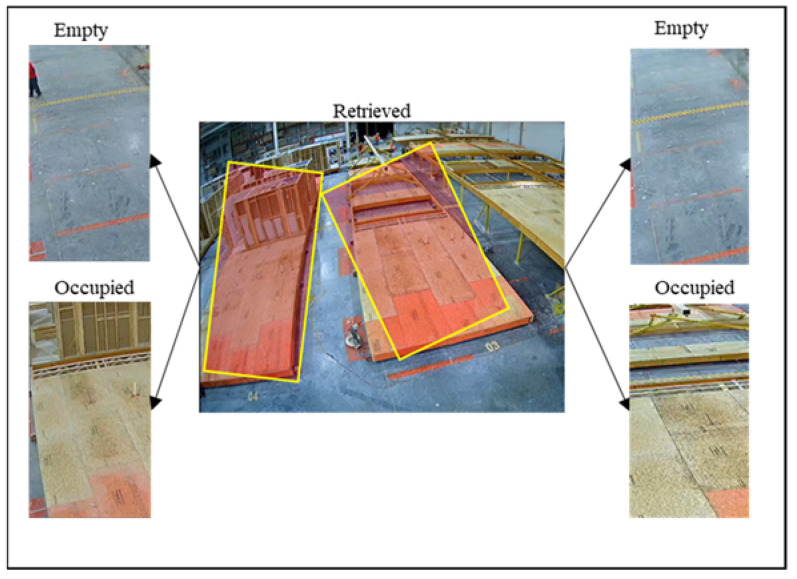
Four ground truth images annotated for two stations.

As shown in Figure 11, the videos covered seven stations which started from the floor preparation at station three and ended with a wire push-up at station nine. The term ‘module’ in this paper is defined as a fully-enclosed building module that consists of a floor, walls, ceiling, and any other component within. In this paper, components such as walls and bath pods are considered part of a modular unit if they are installed on a floor component. For example, in station 3, a bath pod is installed on the floor component, and in the subsequent stations, different wall elements are incrementally assembled and will be considered as parts of a single modular unit after installation. The collected videos contained five days of 12-h shifts with a resolution of 2592 × 1944 pixels, captured at the rate of 12 frames per second (fps). Collected videos were manually annotated as shown in Figure 12.

As shown in Figure 12, ground truth images were manually annotated for each Region of Interest (RoI) only once, resulting in 14 ground truth images for seven stations, and made ready for extracting the features. 

#### 4.1.2. Feature Extraction

**Figure 13:**
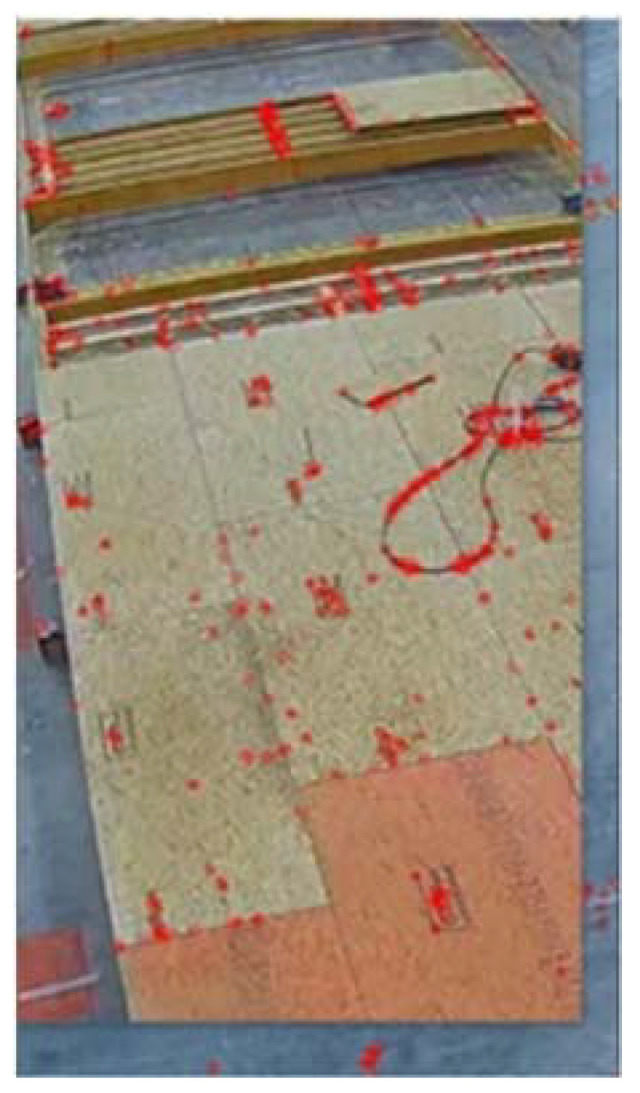
SIFT output where red circles indicate the SIFT keypoints.

SIFT detector and descriptor were used to extract 600 keypoints for each RoI and to describe the keypoints with vectors of size 128. Here, the number of layers at each octave indicates the number of down-sampled images at each octave of the scale space. This parameter was set to three based on the size of the image and the relatively poor quality of the CCTV video footage. The contrast threshold parameter was set to 0.04 to filter out weak features during the feature detection process; however, retain at least 600 keypoints for the images with fewer features, such as the images from the empty state of stations. The edge threshold parameter was set to 10 to filter out the keypoints that are located on the edges. The sigma parameter determines the scale of the Gaussian kernel used to create the scale-space representation of the input image. This parameter was set to 1.6 to pick smaller features as the video footage covers a large area in perspective view, and parts of the modular unit are relatively far from the camera. Figure 13 shows the keypoints with red circles for a sample RoI in station three. 

As shown in Figure 13 and discussed in the discussion section, the proposed method is robust to the presence of some objects with respect to the ground truth, such as a wire in this case; however, the presence of some objects may cause miss classifications as will be discussed in the limitation section. 

#### 4.1.3. Image Matching and Noise Reduction

**Figure 14:**
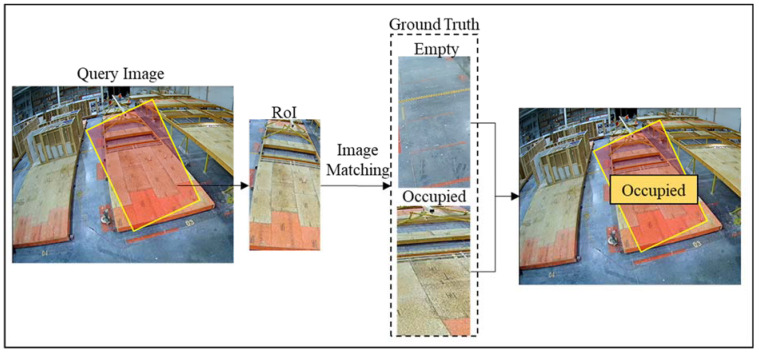
Overview of the image matching pipeline applied on a sample video frame.

Figure 14 shows the image-matching process implemented on the video footage captured from the first camera. 

**Figure 15:**
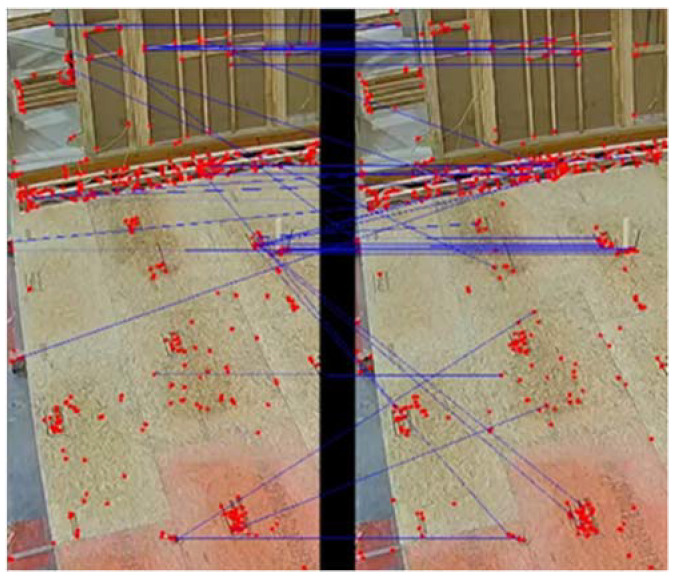
Matching the query frame, on the left, with ‘occupied’ ground truth on the right.

**Figure 16:**
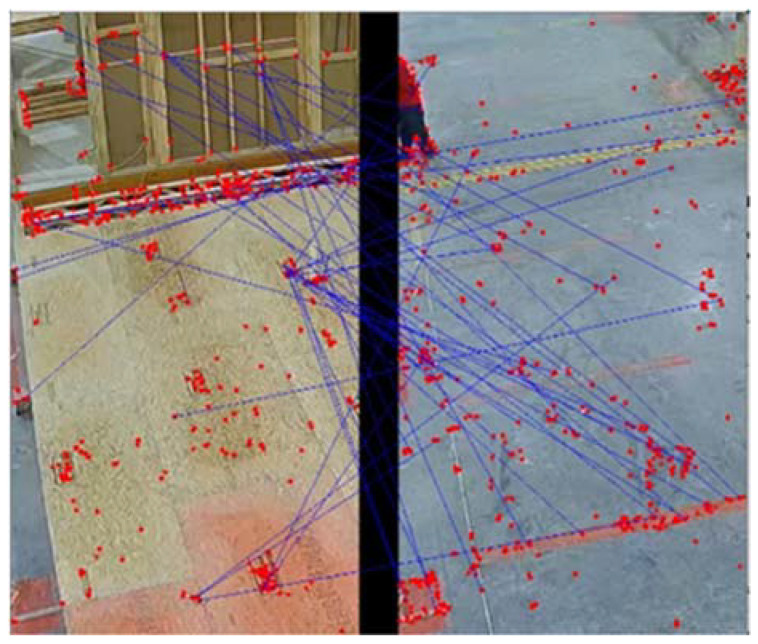
Matching the query frame, on the left, with the ‘empty’ ground truth on the right.

As shown in Figure 14, image matching is performed for each region of interest. Here, each RoI is separately matched with two ground truth images, and the cost of matchings is compared. The smaller cost is used to assign the occupied or empty state to each RoI, and finally, the results from the matching are denoised using the Median Filter method with a kernel size of 15. Figure 15 and Figure 16 show the matching process in more detail, where the query image is compared to the ground truth.

**Figure 17:**
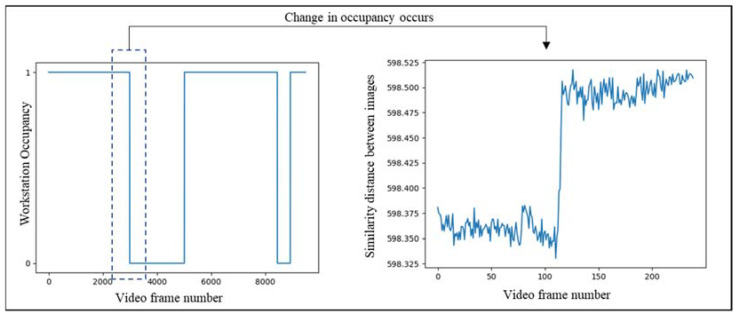
Change in the cost of matching indicating a change in the state of the station.

In Figure 15, the two images are similar, and the majority of the red keypoints are matched properly with their pair in the other image. The transition in the state of the workstation leads to a change in the cost of matching between the pairs of query and ground truth images; therefore, in Figure 16, keypoints are matched with weak keypoints indicating the transition of state in the station. Figure 17 shows the change in the cost of matching as the station becomes empty. 

### 4.2. Workstation State Identification 

**Figure 18:**
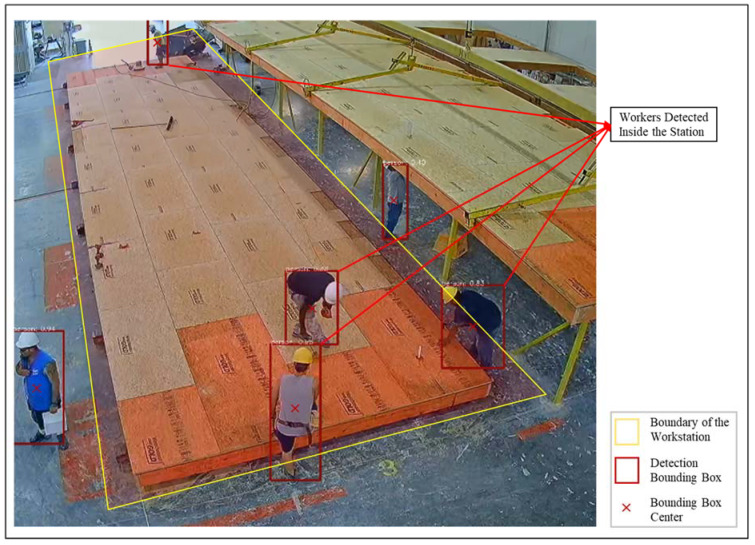
Workers detected inside the workstation in a sample video frame.

In order to identify the presence of the workers, the Mask R-CNN algorithm was pre-trained on the COCO dataset to detect and localize the workers in the stations in the collected videos. Since the pre-trained Mask R-CNN provides high confidence scores for each worker, finetuning was not required; however, the threshold for intersection over union was adjusted in order to increase the precision. Figure 18 shows a sample result where workers are detected and annotated with a bounding box. 

As shown in Figure 18, four of the six detected workers are considered to be inside the station since the center of the detection bounding box falls inside the manually annotated boundary of the workstation. In this case, since workers were present inside the station for the majority of the one-minute window of observation, the station was considered active. 

## 5. Results 

This section provides the results of the developed method in terms of workstation state identification, workstation occupancy identification, and bottleneck detection. 

### 5.1. Workstation Occupancy Identification 

**Figure 19:**
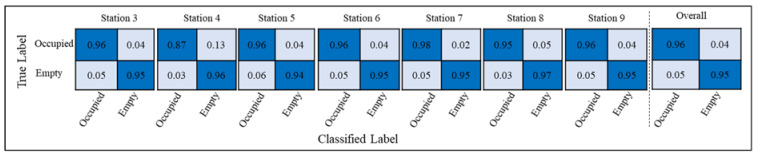
Overall and per-station confusion matrices.

The goal of this method was to timestamp when modular units enter and leave the station and extract the cycle times from the videos. The results from the implementation of the proposed method were compared to the manual analysis of the video to validate the proposed workstation occupancy identification method. The manual analysis was performed by the authors using the ‘Stop Watch’ method. In this method, the videos were observed at a fast pace, the entrance and departure of modular units were timestamped, and the cycle time was extracted.During the observed duration, five modules with the identical design were scheduled to be manufactured. Python OpenCV library was used to label all frames and store them in an Excel sheet. Labeling was conducted for all stations during five days of 12-h shifts to create the ground truth file. Finally, the performance of the method was evaluated for each station. Table 2 provides the evaluation results for the workstation occupancy identification using the accuracy metric, and Figure 19 shows the confusion matrix for each station. 

As shown in Table 2, the developed method provides a high overall accuracy of 96% in identifying the occupancy of workstations when compared to the ground truth. Figure 19 shows that the performance of the proposed method is consistently high within each station and across the production line in the collected videos. This indicates that the proposed method is robust to the changes in the appearance of the modular unit that occur inside each workstation and across the production line as the appearance of the modular units changes from the floor in station three to a complete modular unit in station nine. Table 3 shows how the timestamped data was used to estimate the cycle time of workstation three during five days of work. 

Table 3 shows that the cycle times are significantly variable, which is due to waiting for the subassembly stations; therefore, the active duration can provide decision-makers with fine-grained information about the progress of work, which is empirical for applying improvements to the processes. 

### 5.2. Workstation State Identification 

The goal of this method was to timestamp when occupied workstations are active or inactive. A manual analysis of the videos was conducted to evaluate the performance of the proposed method. Considering the length of the videos, for each station, 100 frames were randomly selected using a Python random number generator library, leading to a total of 700 class-balanced frames. The presence and absence of the workers inside the station in each frame were manually observed, labeled as ground truth, and compared to the results from the vision-based worker detection method. Frames where the worker was present inside the station and was detected by the vision-based method, were marked as true positives, whereas the frames with false worker detection were marked as false positives. False negatives corresponded to frames where a worker was present but not detected by the vision-based method due to a confidence level below the threshold or occlusions. The precision, recall, and F-1 score for this method were calculated using Equations (3)–(5).

(3) $$Precision=TP/\left(TP+FP\right)$$

(4) $$Recall=TP/\left(TP+FN\right)$$

(5) $$F1\;Score=2*\left(Precision*Recall\right)/\left(Precision+Recall\right)$$

**Figure 20:**
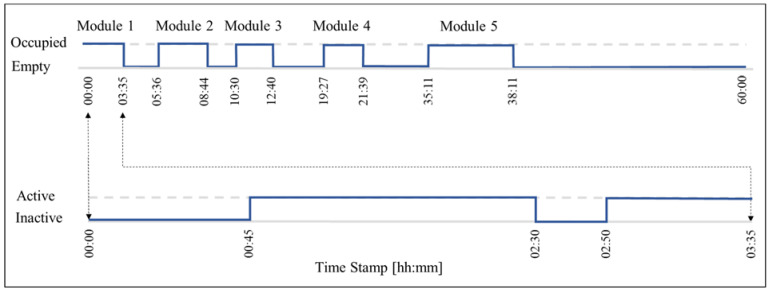
Active periods of workstation three during production of the first module.

Calculating the performance of the proposed vision-based workstation state identification method led to 88% precision, 91% recall, and an 89% F-1 Score. The precision is smaller than the recall due to the larger number of false negatives caused by the occlusions commonly caused by walls, bath pods, and other components. Figure 20 demonstrates the application of the proposed method in station 3 by extracting the cycle times and active times. 

As shown in Figure 20, the cycle times of five modular units are plotted on the top, and the active duration for the first cycle time is on the bottom. Here, five modular units pass through station three within five days of twelve-hour shifts.

The cycle time for the first modular unit in workstation three is 3:35 h, which includes two active durations from 00:45 to 2:30, and from 2:50 to 3:35, resulting in a total active duration of 150 min and an average active duration of 75 min. Monitoring the progress of work during five days of work results in an active duration of 50 min for station three, as shown in Table 4 in the next section. 

### 5.3. Bottleneck Detection 

**Figure 21:**
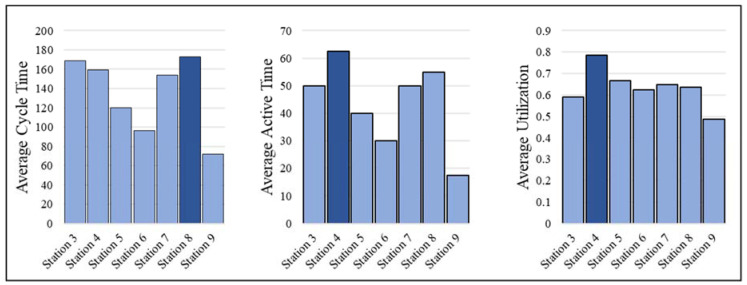
Comparison of metrics extracted using the proposed automated monitoring method.

The goal of this method was to use the proposed automated progress monitoring method to detect the bottleneck station. Table 4 and Figure 21 show the measured cycle time, average active time, and utilization over the five days of production for all stations using the proposed automated monitoring method. Here, the utilization is calculated for further analysis by dividing the observed active period by the cycle time for each station, and average utilization is the average of the calculated utilizations for the entire video.

As shown in Figure 21, workstation eight has the largest average cycle time over five days of work. However, the cycle time standard deviation shown in Table 4 indicates that these cycle times are highly variable and possibly include a large number of inactive periods. The utilization diagram shows that the majority of the stations function with less than 65% utility and include a large number of inactive periods. Station four has the largest utilization indicating this station is more active during its cycle time compared to other stations. Furthermore, this station has the largest average active time, indicating this station is also less likely to be interrupted by other stations. Therefore, station four is the bottleneck station of the observed portion of the production line. This result was also validated using expert opinion from the factory, as this station relies on multiple subassembly stations to provide the side walls, marriage wall, and bath pod. 

## 6. Discussion

This study aimed to propose an automated vision-based method to identify the bottleneck station inside modular construction factories. In this section, the results are further interpreted, and the strengths and weaknesses of the proposed method are discussed within the context of the case study and implications for other factories.

### 6.1. Workstation State Identification

This method was able to identify the empty/occupied stations on the assembly line of modular construction factories with 96% accuracy, using a traditional computer vision technique, simple template-based matching, and user involvement. The high accuracy of 96% shows that this method can be reliably used to identify the occupancy of stations in the current case study. Furthermore, the simple template-based matching expands the generalizability and practicality of this method to other factories by allowing the domain experts to easily annotate two templates for each station in the new factory. This approach also made this method robust to the changes in the appearance of the modular unit, which occur across the production line as the modular units are developed. Finally, the traditional SIFT method reduced the complexity of computations compared to the state-of-the-art machine learning-based computer vision methods.

**Figure 22:**
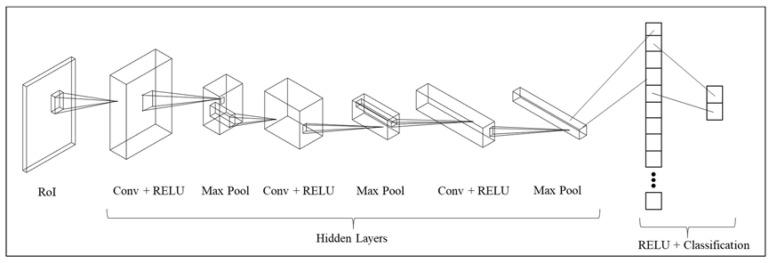
Architecture of the CNN-based binary classification mode used for comparison purposes.

Due to the recent success of machine learning-based computer vision methods, here, the SIFT method is juxtaposed with a CNN-based image classification method in terms of performance, data efficiency, and computational complexity. Figure 22 shows the high-level architecture of the CNN model used for this purpose. 

As shown in Figure 22, the CNN model comprises three CNN layers, with three max-pooling layers and a fully connected layer. The model was trained using a class-balanced dataset of 80 images, including images of occupied and empty stations at different stages of the production; Grid search was used to optimize the hyperparameters; Adam, SGD, and AdaBoost optimizers were considered where Adam outperformed the alternatives; ReLU Activation function outperformed Sigmoid; Images were normalized and resized, and the model was trained for 100 epochs tuned with the learning rate of 0.001. Finally, the model was tested on the same dataset used to evaluate the overall performance of the SIFT method, and the model achieved 91.1% accuracy. 

Although the performance of the CNN model is close to the performance of the SIFT method, the CNN method is an overkill for this task since the SIFT method achieves even slightly higher performance with more data efficiency. In general, algorithms such as SIFT are not class-specific; that is, they are very general and perform the same for any image [47]. In contrast, features learned from a deep neural net are specific to the training dataset; that is, a significant annotation effort is required to achieve the same goal for an assembly line of modular units with different designs, materials, and even later on in the production line where the modules are wrapped with protective plastic and are ready for shipment. Although directly incomparable, currently, traditional methods such as SIFT (with computational complexity of O(ij + k) number of instructions for an image size of I × j and k keypoints) and SURF are more computationally efficient compared to state-of-the-art algorithms such as Mask R-CNN (7 billion number of floating-point operations per image with Resnet-101 backbone). 

### 6.2. Workstation Occupancy Identification

Here, the active/inactive states were identified for the stations. Due to the labor-intensive nature of the tasks, the presence of the workers was directly correlated with these states. The Workstation Occupancy Identification method uses a popular person detection algorithm to detect the workers inside a manually defined boundary. This method achieved a high precision, recall, and F-1 score of 88%, 91%, and 89%. Analysis of the results further unveiled that the failed cases were commonly related to the performance of the model and the occlusions caused due to the oblique view of the CCTV video camera and installation of new components such as walls and bath pods. Although it is not the focus of this study, the model can be fine-tuned on the images of workers, or better-performing models can be used to improve performance. CCTV video footage from different views can be used and integrated into the current view using multiplex techniques to overcome the occlusion problem.

### 6.3. Bottleneck Detection

Finally, this study used a data-driven bottleneck detection method to identify the bottleneck station using the active/inactive durations. This approach has been previously investigated in highly automated factory settings. However, extracting these parameters in real-time is challenging for labor-intensive factories such as the modular construction factory in this study. Therefore, most previous bottleneck detection methods inside modular construction factories relied on manually observed data. The novelty of this study is related to applying this method in manually-intensive factories such as modular construction factories where the active/inactive durations are not provided by the machine itself and require a novel monitoring method, such as the proposed vision-based monitoring method, to extract these durations. As shown in the results section, this method successfully identified the bottleneck station when validated with the factory expert’s opinion. 

## 7. Conclusions

This paper proposed a method to automatically identify the bottleneck station in modular construction factories using computer vision. The developed method extracts the cycle times using traditional computer vision-based methods, identifies the active periods using a deep learning-based algorithm, and identifies the bottleneck workstation using the average active method. The method was successfully validated on videos collected from a modular construction factory in the U.S.

The study contributes to the body of knowledge and practice in the following ways: (1) The method overcomes drawbacks of contact sensor-based methods by proposing a vision-based approach to automatically identify the bottleneck station inside modular construction factories; (2) The method provides a useful insight for factory management by simultaneously processing data from multiple stations; (3) The method is robust to significant changes in the appearance of the modular units across multiple stations and can be used in different stages of production. This was achieved by proposing a per-station classification approach; (4) This study shows how the extracted information from the proposed monitoring method can be used to identify the bottlenecks using data-driven methods, while the previous research overlooked the application of vision-based monitoring methods in identifying the bottlenecks; (5) The proposed method is adaptable to changes in the layout of the factory. This was achieved by including human intervention during the annotation process at the operational level; (6) The method identifies the duration and frequency of idle time during each cycle time and considers how this affects the bottleneck station, and (7) The proposed method is more practical compared to the previously proposed methods. This method relies on commonly available CCTV video footage and integration of traditional and deep learning methods, which provides intuition to the factory experts during the validation of results and reduces the cost of annotation as it requires significantly a smaller number of annotations compared to deep learning-based alternatives. Therefore, the successful implementation of this method can lead to continuous and comprehensive monitoring of the production line and preventing delays by timely identification of bottlenecks. 

Limitations of the proposed method were identified by analyzing failed cases. The workstation occupancy identification method misclassified a few frames with a greater number of workers in the image, which reduced the accuracy of this method, as shown in Table 2. Furthermore, in the proposed state identification method, the presence of workers is directly associated with the active state of the workstation. This assumption can be violated in cases where workers are present at an occupied workstation but are not working. Lastly, the scope of this study is limited to buffer-less assembly lines and does not take into account the safety stocks that form between offline stations. 

Future work can focus on addressing the limitations of the proposed method. To address the challenges related to workstation occupancy identification, an ensemble of multiple computer vision-based methods can be used to identify the occupancy of the workstation, and the majority voting method can be used to increase the confidence level of the results. To address the limitations of the workstation state identification method, human activity recognition methods can be used to classify the activity of the workers and automatically discard instances where the workers are not working. Specifically, experiments will be performed using both spatial and spatial-temporal action segmentation and activity recognition models. Within this framework, a combination of close-range cameras and CCTV cameras can be used to retrieve videos with higher resolution at each station for activity recognin and use CCTV footage for higher-level progress monitoring over the entire factory. Finally, this framework can be generalized to the entire factory by monitoring the buffers and safety stocks between offline stations and identifying the bottleneck for the entire factory. 

## Figures and Tables

**Table 1:** Instances where the workstation is active or inactive in modular construction factories.

Station	Example
Active	Work is being performed at the station such as installation, finishing, nailing
Inactive	Station is occupied by volumetric unit, but is waiting for other parts such as the wall frame

**Table 2:** Per-station and overall performance of workstation occupancy identification.

Station	Station 3	Station 4	Station 5	Station 6	Station 7	Station 8	Station 9	Overall
Accuracy	0.96	0.93	0.94	0.95	0.96	0.96	0.96	0.96

**Table 3:** Cycle times at workstation three. Times are in [hh:mm] format.

Modular Unit ID	Start	Finish	Cycle Time
1	00:00	3:35	03:35
2	05:36	08:44	03:08
3	10:30	12:40	02:10
4	19:27	21:39	02:12
5	35:11	38:11	03:00

**Table 4:** Progress monitoring report using the proposed progress monitoring method.

Metric	Station 3	Station 4	Station 5	Station 6	Station 7	Station 8	Station 9
Average Cycle Time (min)	169	159	110	96	154	173	72
Average Active Time (min)	50	62.5	40	30	50	55	17.5
Average Utilization (%)	59	78	66	62	65	63	48
Cycle time STD (min)	37	43	7	3.5	19	9	17

## Data Availability

Not applicable.
